# Does Intrauterine Injection of hCG Improve IVF Outcome? A Systematic Review and a Meta-Analysis

**DOI:** 10.3390/ijms232012193

**Published:** 2022-10-13

**Authors:** Alessandro Conforti, Salvatore Longobardi, Luigi Carbone, Giuseppe Gabriele Iorio, Federica Cariati, Maria Rosaria Campitiello, Ida Strina, Michela Palese, Thomas D’Hooghe, Carlo Alviggi

**Affiliations:** 1 Department of Neuroscience, Reproductive Science and Odontostomatology, University of Naples Federico II, 80131 Naples, Italy; 2 Merck Serono S.p.A, 00176 Roma, Italy; 3 Department of Obstetrics and Gynecology and Physiopathology of Human Reproduction, ASL Salerno, 84124 Salerno, Italy; 4 Department of Development and Regeneration, Biomedical Sciences Group, KU Leuven (University of Leuven), 3000 Leuven, Belgium; 5 KGaA, 64293 Darmstadt, Germany

**Keywords:** IVF, ICSI, embryo implantation, intrauterine administration, hCG, ART

## Abstract

Various interventions have been proposed to improve embryo implantation in IVF. Among these, intrauterine injections of human chorionic gonadotropin seem to have promising results. Consequently, we conducted a review and meta-analysis to assess IVF outcomes by comparing couples who underwent intrauterine hCG injection transfer versus those who underwent embryo transfer with intrauterine injection of placebo, or without any additional intervention. The primary outcome was the clinical pregnancy rate. Secondary outcomes were the implantation rate, miscarriage rate, and live birth rate. A meta-analysis was conducted using the random effects model, while bias within studies was detected using the Cochrane risk of bias tool. Ectopic pregnancies and stillbirths were also assessed. The clinical pregnancy (RR 1.38, 95% CI 1.17–1.62, *p* < 0.0001) and implantation rate (RR 1.40, 95% CI 1.12–1.75, *p* = 0.003) were significantly higher in women who underwent hCG injection than in the control group. These significant effects persisted only in women who underwent cleavage-stage embryo transfer. No significant differences between groups were observed in the other secondary outcomes. In conclusion, our systematic review and meta-analysis demonstrate that intrauterine injection of hCG could be a valuable approach in women who undergo cleavage-stage embryo transfer. Given the lack of data about the live birth rate, caution should be exercised in interpreting these data.

## 1. Introduction

Embryo implantation is a crucial process in assisted reproduction. It involves a complex process between the endometrium and the implanted embryo that consists of three stages: apposition, adhesion, and invasion [1]. From a clinical perspective, successful implantation occurs when a gestational sac is seen on ultrasonographic imaging. Unfortunately, human embryo implantation is relatively inefficient. In fact, it was estimated that approximately 75% of pregnancy losses are due to defective implantation [2,3]. In addition, several other factors may interfere with embryo implantation. For instance, exposure of embryos to the culture media or artificial manipulation of the endometrium during embryo transfer could affect the interaction between the endometrium and the embryo [4]. Furthermore, supraphysiological levels of steroids recorded during conventional ovarian stimulation could negatively affect oocyte maturation and endometrium development [4]. Given the poor efficiency of embryo implantation, various interventions, particularly in the IVF context, have been proposed to improve this process [5,6,7,8,9]. Among these, intrauterine injections of human chorionic gonadotropin (hCG) seem to have promising results [10,11]. The rationale for using hCG to improve embryo implantation is supported by both animal and in vivo studies. Indeed, hCG, by modulating factors involved in embryo implantation (i.e., endometrial matrix-metalloproteinases, growth factors, and cytokines), could improve endometrial receptivity [12,13]. In addition, there is evidence that hCG exerts a pivotal paracrine role during embryo implantation [14].

Various randomized controlled trials (RCTs) and meta-analyses have investigated the effect of intrauterine injection of hCG before embryo transfer in women undergoing IVF [15,16,17,18,19,20,21]. The most recent Cochrane review includes 17 RCTs, 6 of which are conference abstracts, and concluded that women undergoing cleavage-stage transfer might benefit from intrauterine hCG [20]. Since the publication of the latter paper, another seven RCTs have been published [22,23,24,25,26,27,28]. More recently, a comprehensive meta-analysis of different interventions, including intrauterine hCG during embryo transfer, concluded that intrauterine hCG could significantly increase the clinical pregnancy rate [29].

Given the relevance of the topic and recent results [25,26,28], it is pertinent to update previous data. Consequently, we conducted a review and meta-analysis to assess IVF outcomes by comparing couples who underwent intrauterine hCG injection transfer versus those who underwent embryo transfer with intrauterine injection, placebo, or without any additional intervention. Unlike previous meta-analyses on this topic [21,29], we did not consider conference abstracts in our study because they often lack details about study design, methods, risk of bias, and outcomes [30]. Furthermore, given the impact of embryo culture duration on embryo implantation [31], we distinguished cleavage-stage data from data on blastocyst embryo transfer. Lastly, we investigated the effect of intrauterine hCG on women who experienced implantation failure.

METHODS

We adhered to PRISMA guidelines [32,33]. The study protocol was registered at PROSPERO(registration number CRD42022300563) on February 2022.

### 1.1. Search Strategy

We searched the MEDLINE (PubMed), ISI WEB OF KNOWLEDGE, SCOPUS, and EMBASE databases up to April 2022. We also searched the reference lists of relevant studies and reviews. Combinations of the following keywords and search terms were used: (“implantation failure” OR “repeated implantation failure” OR “recurrent implantation failure” OR “implantation” OR “implantation rate”) AND (“intrauterine” OR “intrauterine device” OR “intrauterine administration” OR “intrauterine infusion” OR “Intrauterine HCG).

### 1.2. Study Selection

We used the Population, Intervention, Comparison, and Outcomes (PICO) model to select our study population. In detail, we included only RCTs in which women underwent in vitro fertilization and embryo transfer (Population). Intrauterine hCG injection before embryo transfer (Intervention) was compared with placebo or control with no intrauterine intervention (Control). Further details are reported in Supplementary Table S1. No time or language restrictions were adopted, and queries were limited to human studies. Excluded studies were cohort studies, retrospective studies, case series, case reports, books, congress abstracts, and gray literature.

### 1.3. Study Outcomes

Primary outcome was clinical pregnancy rate, defined as “a pregnancy diagnosed by ultrasonographic visualization of one or more gestational sacs or definitive clinical signs of pregnancy”. Secondary outcomes were implantation rate (the number of gestational sacs observed divided by the number of embryos transferred), miscarriage rate (the spontaneous loss of an intra-uterine pregnancy prior to 22 completed weeks of gestational age in relation to clinical pregnancy), and live birth rate (delivery rate per initiated cycle). Adverse events, namely, ectopic pregnancies (pregnancy outside uterine cavity) and stillbirth (the death of a fetus prior to the complete expulsion or extraction from its mother after 28 completed weeks of gestational age), were also assessed. All study outcomes were consistent with the International Glossary on Infertility and Fertility Care [34].

### 1.4. Data Extraction

Two authors (A.C. and L.C.) evaluated titles and abstracts. Duplications were removed using Endnote online software and manually. Data were extracted using predefined data fields. In detail, we developed a data-extraction sheet based on the Cochrane data-extraction template. Data were extracted independently by two reviewers (A.C. and L.C.), and discrepancies were resolved by discussion with the most experienced authors (C.A., S. L., and T.D.). When important information was lacking in the original publications, we contacted the authors.

### 1.5. Assessment of Both the Risk of Bias and Publication Bias

Two authors (A.C. and M.C.) independently assessed the risk of bias in the studies eligible for the review using the Cochrane risk of bias tool [35]. The following issues were assessed: (1) random sequence generation; (2) allocation concealment; (3) blinding of participants and personnel; (4) incomplete outcome data; (5) selective reporting; (6) other bias. For each issue, the risk of bias was graded low, unclear, or high. Publication bias was assessed using the funnel plots of primary outcome both visually and formally using the trim-and-fill method [36]. This evaluation was performed using Prometa 3.0 software. 

### 1.6. Statistical Analysis

Statistical analysis was carried out using the RevMan software (The Nordic Cochrane Centre, The Cochrane Collaboration. Review Manager version 5.4). Categorical data were combined to obtain a pooled risk ratio (RR). A meta-analysis was conducted using the random effect model. Between-study heterogeneity was addressed using I^2^, which represents the percentage of total variation in the estimated effect across studies. An I^2^ value over 50% indicates substantial heterogeneity. *p* values below 0.05 were considered statistically significant.

### 1.7. Subgroup Analysis

We conducted a subgroup analysis to separate women who underwent embryo transfer at cleavage stage from those who underwent embryo transfer at blastocyst stage. Primary outcome in women who experienced implantation failure was also explored.

## 2. Results and Discussion

### 2.1. Study Selection and Characteristics

**Figure 1:**
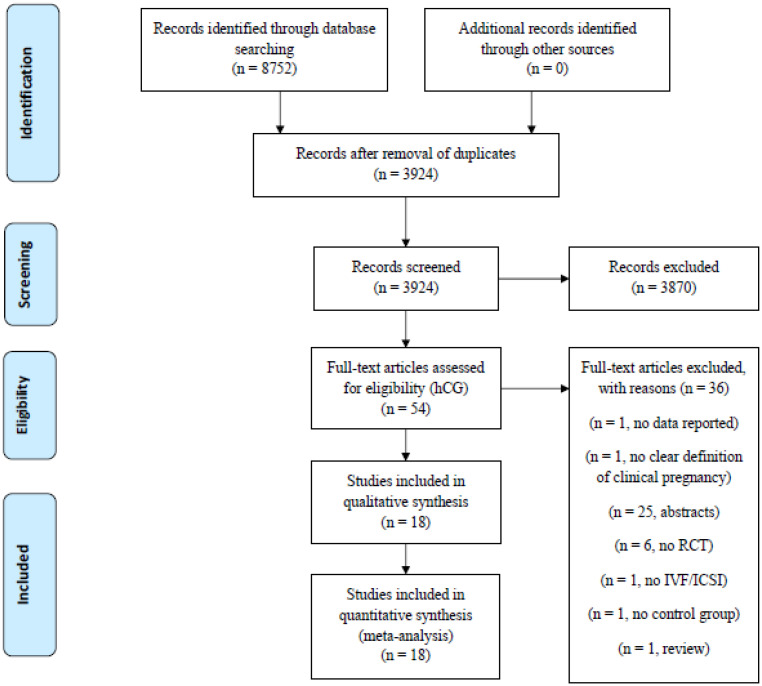
Study flow chart according to PRISMA guidelines.

A total of 8752 papers were identified in MEDLINE (PubMed), the ISI WEB OF KNOWLEDGE, SCOPUS, and EMBASE (Figure 1). Duplications were removed by Endnote Online and manually. Fifty-four papers were assessed for eligibility. Eighteen RCTs were included in the qualitative and quantitative analysis [10,11,15,16,17,18,22,23,24,25,26,27,28,37,38,39,40,41]. The baseline characteristics of the studies included are reported in Table 1.

### 2.2. Risk of Bias within and across Study

Random sequence generation was conducted appropriately in 17 out of 18 studies. Allocation concealment was conducted with a low risk of bias in 6 studies, while uncertain risk and high risk of bias were detected in 10 and 2 studies, respectively. Most studies had a low risk of bias in terms of the blinding of participants and personnel (11 out of 18), while a high risk of bias and uncertain risk of bias was detected in 6 and 1 studies, respectively. Only two studies were classified as having an uncertain risk of bias regarding the blinding of outcome assessment. A high risk of bias for incomplete outcome was observed in four studies due to the loss of patients’ follow-up, while uncertain risk was observed in three studies. The majority of RCTs were classified as having an unclear risk of selective reporting bias because no data about the live birth rate were reported (12 out of 18 studies). Nonetheless, 17 out 18 studies reported data concerning primary outcomes. Considering the interim analysis and the change of study protocol, the study by Mansour et al. 2011 [39] was considered to be at high risk of other sources of bias.

Further details are reported in Supplementary Figure S1. No relevant risk of bias across the studies was observed (Supplementary Figure S2).

### 2.3. Summary of Findings

#### 2.3.1. Clinical Pregnancy Rate

**Figure 2:**
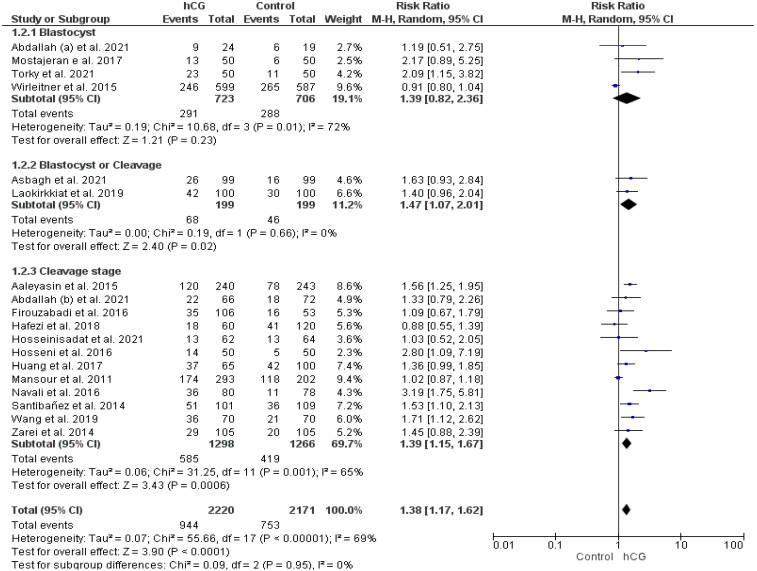
Forest plot showing the effect of intrauterine hCG injection versus the control group on clinical pregnancy rate.

Seventeen papers assessed the clinical pregnancy rate (total participants = 4391). The clinical pregnancy rate was significantly higher in women who underwent hCG injection than in the control group (RR 1.38, 95% CI 1.17–1.62, I^2^: 69%, *p* < 0.0001). In the subgroup analysis of the duration of embryo culture, this significant effect persisted only in women who underwent cleavage-stage embryo transfer (RR 1.39, 95% CI 1.15–1.67, I^2^: 65%, *p* = 0.0006) (Figure 2). Only four studies investigated the effect of hCG in women with a history of recurrent implantation failure. Among them, only one RCT included women with recurrent implantation failure consistent with ESHRE criteria [28]. A significantly higher clinical pregnancy rate was observed in women who underwent hCG intrauterine injection versus controls (RR 1.56, 95% CI 1.26–1.94, I^2^: 0%, *p* < 0.0001) (Supplementary Figure S3).

#### 2.3.2. Miscarriage Rate

**Figure 3:**
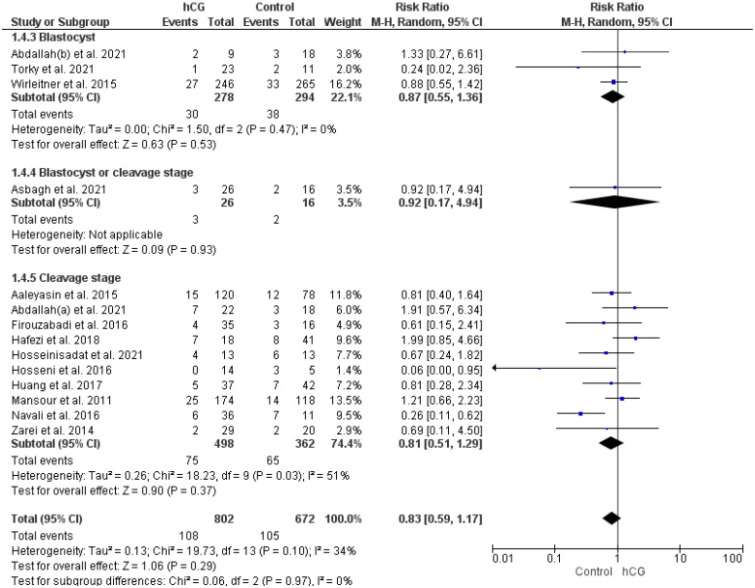
Forest plot showing the effect of intrauterine hCG injection versus control group on miscarriage rate.

Thirteen studies investigated the miscarriage rate (pregnancies = 1474). A comparable miscarriage rate was observed in the two groups, irrespective of embryo culture duration (Figure 3).

#### 2.3.3. Implantation Rate

**Figure 4:**
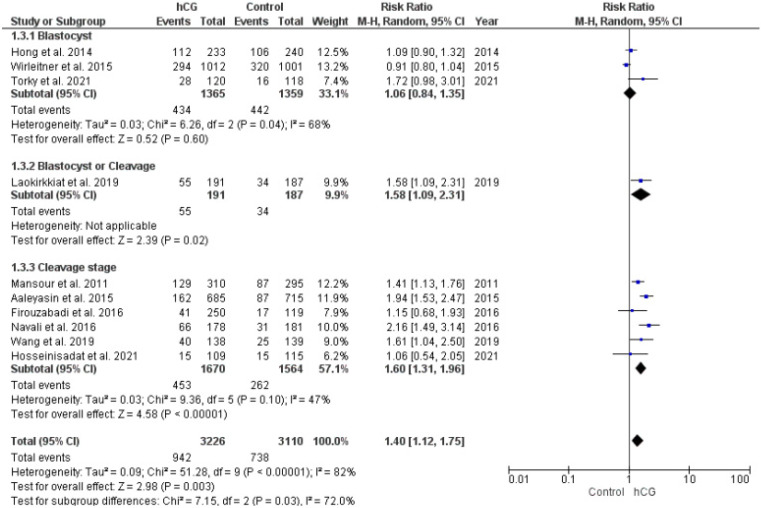
Forest plot showing the effect of intrauterine hCG injection versus control group on implantation rate.

Ten studies evaluated the implantation rate (embryo transferred = 6336). Overall, the implantation rate was better in women who underwent hCG intrauterine injection than in the control groups (RR 1.40, 95% CI 1.12–1.75, I^2^: 82%, *p* = 0.003). In the subgroup analysis, according to the duration of embryo culture, a significant effect persisted only in women who underwent cleavage-stage embryo transfer (RR 1.60, 95% CI 1.31–1.96, I^2^: 47%, *p* < 0.00001) (Figure 4).

#### 2.3.4. Live Birth Rate

**Figure 5:**
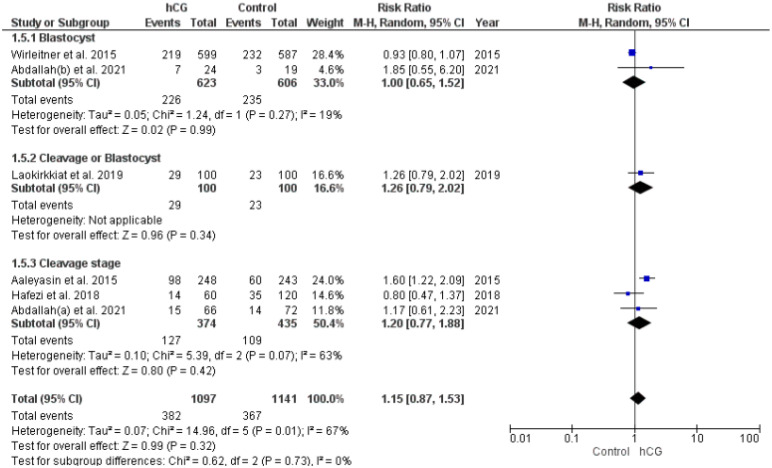
Forest plot showing the effect of intrauterine hCG injection versus control group on live birth rate.

Five studies reported data concerning the live birth rate (total participants = 2238). The live birth rate was comparable in the two groups (Figure 5).

#### 2.3.5. Ectopic Pregnancy and Stillbirth

**Figure 6:**
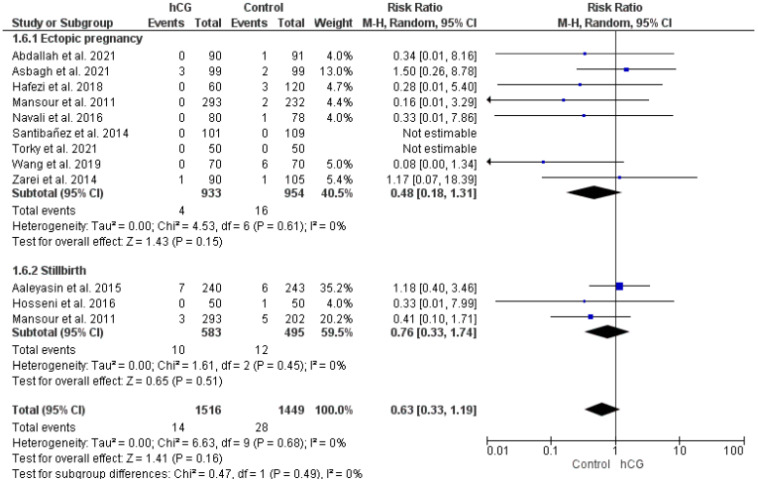
Forest plot showing the effect of intrauterine hCG injection versus control group on adverse events (ectopic pregnancy, stillbirth).

Ectopic pregnancy and stillbirths were reported in nine and three studies, respectively. The occurrence of these two adverse events was similar in the two groups (Figure 6).

### 2.4. Synthesis of Results

#### 2.4.1. Summary of Evidence

The systematic review and meta-analysis of RCTs demonstrate that intrauterine injection of hCG leads to a better outcome in IVF in terms of the clinical pregnancy rate and implantation rate versus a control group. In contrast, the live birth rate and miscarriage rate were similar in the treated and untreated groups. We believe that the discrepancies between live births and the clinical pregnancy rate is related to the different number of participants and studies included in the study. Indeed, the clinical pregnancy rate was assessed in most of the RCTs evaluated for a total of 18 studies and 4391 participants. Conversely, the live birth rate was assessed in only 5 studies for a total of 2238 participants. Notably, the clinical pregnancy rate is considered a reliable parameter of IVF success [42], so the clinical benefit of intrauterine hCG injection appears to be possible anyway. This benefit is mainly linked to the crucial effects that hCG exerts during embryo implantation. Indeed, several lines of evidence suggest that hCG could promote trophoblast invasion and vascular interaction with intervillous space during the first phases of pregnancy [43,44]. Furthermore, hCG could modulate uterine natural killer functions and could influence complement factor and T cell proliferation, thereby increasing immunological tolerance during embryo implantation [45,46,47]. Lastly, hCG is able to sustain the morphological and functional differentiation of human endometrial stromal cells into decidua [48] and can modulate the expression of prostaglandin and chemokine receptors that are involved in embryo implantation [49]. Our data are consistent with the Cochrane reviews conducted by Craciunas et al. in 2018 [20], thereby indicating that an effect on clinical pregnancy can be seen, especially after cleavage stage embryo transfer. However, compared with the Cochrane reviews, the robustness of our findings is supported by the higher number of cases and RCTs included (18 versus 11 studies). Indeed, the main strength of our meta-analysis is the high number of RCTs included (over 4000 participants involved in the analysis of the primary outcome). Moreover, we have included only full-text papers and excluded abstracts and conference meetings that could be a further source of bias [30].

Why hCG seems to be effective before cleavage-stage embryo transfer and not before blastocyst embryo transfer is still unclear. A possible explanation could be that, in contrast to cleavage-stage embryos, blastocysts could, per se, promote the production of molecular signaling, which is important for embryo implantation [31,50]. However, it seems that hCG is not unnecessary in all women who underwent blastocyst transfer. For instance, in a prospective cohort study, Riboldi et al. observed that hCG injection could improve endometrial receptivity when poor-quality blastocysts are transferred [51]. In addition, Torky et al. observed that hCG injection at the time of oocyte retrieval could improve the implantation rate and clinical pregnancy rate in women with a history of recurrent implantation failure (RIF) who underwent blastocyst embryo transfer [28]. However, whether hCG could be of benefit in these cases requires further investigations.

The dosage of intrauterine hCG that appeared to be most effective is at least 500 UI [39]. Indeed, Mansour et al. demonstrated that the administration of 200 UI or 100 UI is not sufficient to obtain appreciable results in terms of the pregnancy rate [39]. Conversely, at a dosage of 500 UI, the authors observed significantly better implantation and clinical pregnancy rates [39]. All studies included used a formulation at the dose equal to or above 500 UI. Regarding timing, most trials administrated hCG from 3 to 15 min before embryo transfer. Conversely, Navali et al. (2016) and Torky et al. (2021) administrated hCG at the time of ovum pick-up [28,41]. Urinary formulation was the most frequently used; only one RCT adopted recombinant formulation at the dosage of 250 µg (equivalent to 6500 IU) [16].

#### 2.4.2. Limitations

The main limitation of this study is the heterogeneity in terms of formulations and protocols adopted. Consequently, we adopted a conservative approach using the random effects model in our meta-analysis independently of I2 values. One of the main causes of heterogeneity is the absence of a standardized timing regarding when to inject hCG into the uterus. Considering the information that we have collected so far, it seems that this procedure should be carried out a few minutes before embryo transfer. However, the only RCTs that investigated the effect of hCG injection in women with recurrent implantation failure resulted in a significantly better clinical pregnancy rate even if the procedures were performed at the time of ovum pick-up. Similarly, Navali et al. obtained excellent results in 158 women with a normal ovarian reserve and ≤41 years old without a history of RIF. Thus, the appropriate timing of hCG injection is still under debate. Another source of bias could be represented by the fact that, among trials, different culture media were adopted for intrauterine injection. As reported in Supplementary Figure S1, most of the trials did not report data concerning the live birth rate, which is considered the most important endpoint in the IVF context. The main reason behind this issue is the fact that the follow-up of pregnancy until delivery could be difficult and expensive for IVF centers. Even stillbirths, which are a complication beyond 28 weeks of pregnancy, were reported in only 3 RCTs. To overcome this, we have selected as the main endpoint the clinical pregnancy rate, which is considered a reliable endpoint to explore the effectiveness of treatments [42]. In this context, Clarke et al., in a meta-analysis of 143 RCTs, demonstrated that conclusions regarding the effectiveness of a treatment based on either clinical pregnancy or live birth as endpoints are comparable [42]. The fact that 11 out of 18 RCTs had a high risk of bias in at least one of the domains assessed is another limitation of this meta-analysis. The most outstanding issues concern the lack of blinding and incomplete outcome data. Thus, higher-quality. RCTs are required to confirm our results. Notably, another limitation of our study is that a regional bias could not be excluded, given that most RCTs involved women from the Middle East IVF centers (mainly from Iran and Egypt). Unfortunately, we were not able to assess this properly due to a lack of studies involving other ethnic groups.

## 3. Conclusions

Our systematic review and meta-analysis demonstrated that intrauterine injection of hCG could be a valuable approach in women who undergo cleavage-stage embryo transfer. Promising results were also observed in women who experienced implantation failure. The absence of a significant effect on the live birth rate, which may be due to a high rate of reporting bias observed among RCTs included, imposes caution in the interpretation of data and should encourage the development of more robust trials in the future.

## Figures and Tables

**Table 1:** Characteristics of studies included.

Reference	Country	Population	Intervention	Comparators	Embryo Stage
Aaleyasin et al., 2015 [11]	Iran	N = 483 <40 years old	n = 240 500 IU of hCG, 5–7 min before ET	n = 243 50 μL tissue culture media, 5–7 min prior to ET	Cleavage stage
Abdallah et al., 2022 [25]	Egypt	N = 181 18–43 years old, at least one good-quality embryo to transfer	n = 90 hCG (500 IU in 0.1 mL of tissue culture media) 4 min before ET	n = 91 Culture media (0.1 mL)	Clevage stage; Blastocyst stage
Asbagh et al., 2021 [26]	Iran	N = 198 <40 years old, ≥1 implantation failures	n = 99 500 IU of hCG, 15 min before ET	n = 99 No intervention	Clevage stage; Blastocyst stage
Dehghani Firouzabadi et al., 2016 [18]	Iran	N = 159 20–40 years old	n = 106 500 IU hCG, approx 7 min before ET 1000 IU hCG, approx 7 min before ET	n = 53 No intervention	Cleavage stage
Hafezi et al., 2018 [22]	Iran	N = 180 <40 years old, 1st FET and ≥1 implantation failures (fresh IVF/ICSI cycle)	n = 60 500 IU of hCG, 7–10 min before ET	n = 60 40 μL of culture medium, 7–10 min before ET n = 60 No intervention	Cleavage stage
Hong et al., 2014 [10]	USA	N = 300 <43 years old	n = 148 500 IU of hCG, less than 3 min before ET	n = 152 ET media, before ET	Blastocyst stage
Hosseinisadat et al., 2021 [27]	Iran	N = 126 <40 years old	n = 62 1000 IU of hCG	n = 64 No intervention	Cleavage stage
Hosseini et al., 2016 [37]	Iran	N = 100 <40 years old	n = 50 500 IU of hCG, 7 min before ET	n = 50 No intervention	Cleavage stage
Huang et al., 2017 [38]	China	N = 165 ≤38 years old, ≥2 implantation failures	n = 65 1000 IU of hCG, 3 days before ET	n = 50 Physiological saline before ET n = 50 No intervention	Cleavage stage
Laokirkkiat et al., 2017 [23]	Thailand	N = 200 18–43 years old	n = 100 500 IU of hCG, 4 min before ET	n = 100 10 μL of culture medium, 4 min before ET	Cleavage stage; Blastocyst
Mansour et al., 2011 [39]	Egypt	N = 445 <40 years old	n = 243 100 IU of hCG vs. 200 IU of hCG vs. 500 IU of hCG, 7 min before ET	n = 202 No intervention	Cleavage stage
Mostajeran et al., 2017 [40]	Iran	N = 100 20–40 years old	n = 50 700 IU of hCG, 5–10 min before ET	n = 50 No intervention	Blastocyst
Navali et al., 2016 [41]	Iran	N = 158 ≤41 years old	n = 80 500 IU hCG in up to 0.5 mL normal saline, immediately after oocyte retrieval	n = 78 0.5 mL normal saline, immediately after oocyte retrieval	Cleavage stage
Santibañez et al., 2014 [15]	Mexico	N = 210 <40 years old	n = 101 500 IU of hCG, before the ET	n = 109 Same culture media without hCG	Cleavage stage
Torky et al., 2021 [28]	Egypt	N = 100 20–39 years old, ≥3 implantation failures of good quality embryo	n = 50 5000UI c, at the time of ovum pick-up	n = 50 Saline solution (placebo), at the time of ovum pick-up	Blastocyst
Wang et al., 2019 [24]	China	N = 140 Implantation failure definition: (1) embryo transfer + frozen embryo transfer ≥3 transfer cycles; (2) cumulative number of transferred embryos ≥4; (3) each time at least 1 high-quality embryo was transferred	n = 70 500 UI hCG + G2 fluid, 3 min before ET	n = 70 G2 fluid	Cleavage stage
Wirleitner et al., 2015 [17]	Austria	N = 1186 ≤43 years old, ≤2 implantation failure	n = 89 500 IU hCG: 2 days before ET n = 510 500 IU hCG3 min before ET	n = 93 40 μL culture medium: 2 days before ET n = 494 40 μL culture medium 3 min before ET	Blastocyst
Zarei et al., 2014 [16]	Iran	N = 210 18–40 years old	n = 105 250 μg (equivalent to 6500 UI) of recombinant hCG, 12 min before ET	n = 105 Normal saline (0.5 mL), 12 min before ET	Cleavage stage

## Data Availability

Not applicable.

## Supplementary Materials

The following supporting information can be downloaded at: https://www.mdpi.com/article/10.3390/ijms232012193/s1.
