# Supplementary material for: Does Intrauterine Injection of hCG Improve IVF Outcome? A Systematic Review and a Meta-Analysis
Source: Int J Mol Sci. 2022 Oct 13;23(20):12193. doi: 10.3390/ijms232012193 (PMC9603006; doi:10.3390/ijms232012193)
Supplement: Supplementary file 1 [file ijms-23-12193-s001.zip › Supp Table S1 (PICO selection).pdf]

Supplementary Table S1. Selection criteria according to PICO questions

Population	Infertile women who underwent in vitro fertilization and embryo transfer
Intervention	Intrauterine injection of hCG
	versus
Comparison	Placebo or Control with no intrauterine intervention
Outcomes	Clinical pregnancy rate<br>Miscarriage rate<br>Live birth rate<br>Implantation rate<br>Adverse event (ectopic pregnancy, intrauterine death)
Study type	RCT (no abstract included)
