# Supplementary figures and images for: Does Intrauterine Injection of hCG Improve IVF Outcome? A Systematic Review and a Meta-Analysis
Source: Int J Mol Sci. 2022 Oct 13;23(20):12193. doi: 10.3390/ijms232012193 (PMC9603006; doi:10.3390/ijms232012193)

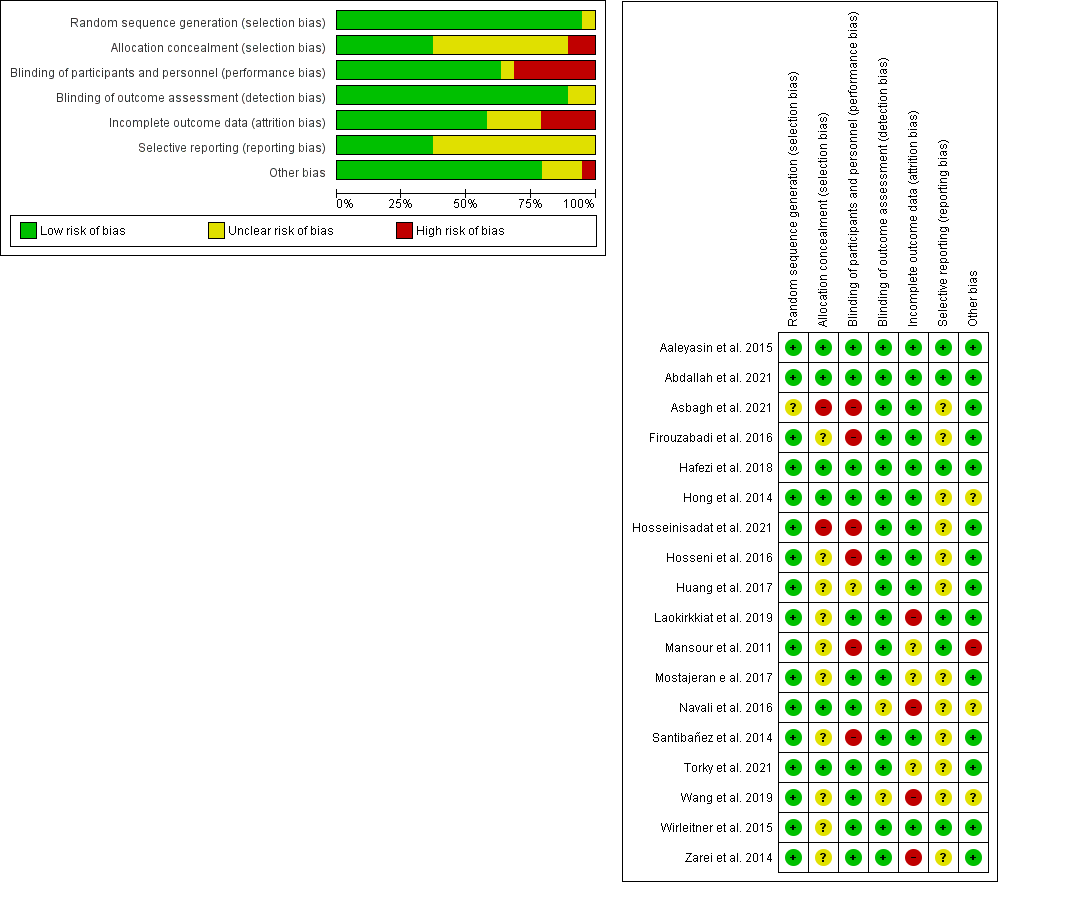

Supplement: Supplementary file 1 [file ijms-23-12193-s001.zip › Supp. Figure S1. Risk of bias graph.png]

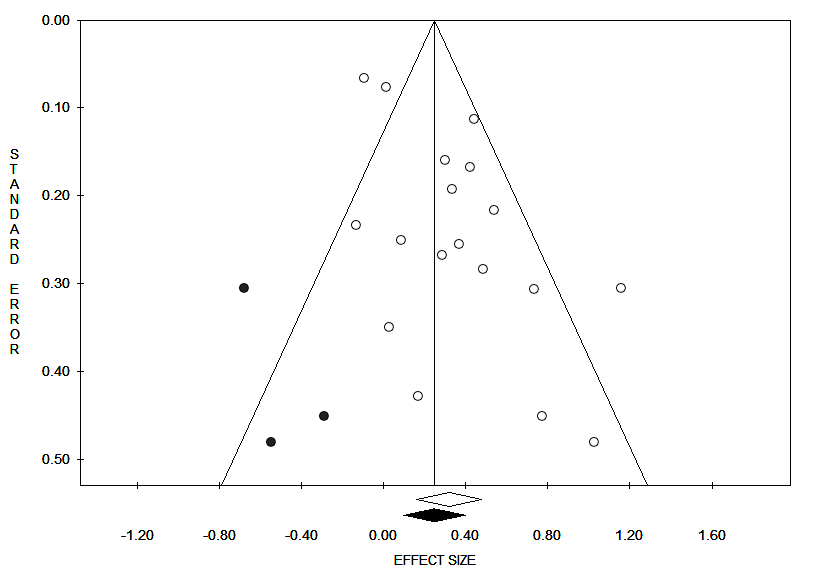

Supplement: Supplementary file 1 [file ijms-23-12193-s001.zip › Supp. Figure S2. Trim and fill figure new.png]

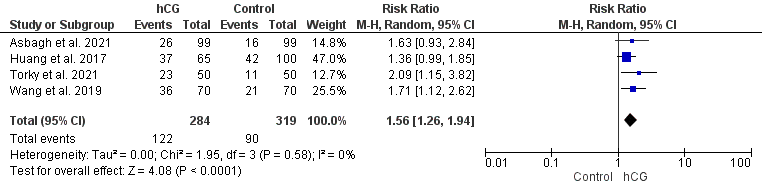

Supplement: Supplementary file 1 [file ijms-23-12193-s001.zip › Supp. Figure S3. Clinical pregnacy rate implantation failure.png]
